# Physicians’ intention to leave direct patient care: an integrative review

**DOI:** 10.1186/s12960-015-0068-5

**Published:** 2015-09-08

**Authors:** Christiane Degen, Jian Li, Peter Angerer

**Affiliations:** Institute of Occupational Medicine and Social Medicine, Centre for Health and Society, Faculty of Medicine, University of Düsseldorf, Düsseldorf, Germany

**Keywords:** Intention to leave direct patient care, Physicians, Medical profession, Integrative review, Physician shortage

## Abstract

**Background:**

In light of the growing shortage of physicians worldwide, the problem of physicians who intend to leave direct patient care has become more acute, particularly in terms of quality of care and health-care costs.

**Methods:**

A literature search was carried out following Cooper’s five-stage model for conducting an integrative literature review. Database searches were made in MEDLINE, PsycINFO and Web of Science in May 2014.

**Results:**

A total of 17 studies from five countries were identified and the study results synthesized. Measures and percentages of physicians’ intention to leave varied between the studies. Variables associated with intention to leave were demographics, with age- and gender-specific findings, family or personal domain, working time and psychosocial working conditions, job-related well-being and other career-related aspects. Gender differences were identified in several risk clusters. Factors such as long working hours and work–family conflict were particularly relevant for female physicians’ intention to leave.

**Conclusions:**

Health-care managers and policy-makers should take action to improve physicians’ working hours and psychosocial working conditions in order to prevent a high rate of intention to leave and limit the number of physicians actually leaving direct patient care. Further research is needed on gender-specific needs in the workplace, the connection between intention to leave and actually leaving and measures of intention to leave as well as using qualitative methods to gain a deeper understanding and developing validated questionnaires.

## Background

Losing physicians from the workforce is a problem in many countries and particularly in those where there is already a physician shortage [1,2]. Research on the physician shortage has found that the causes in many countries are the poor geographical distribution of physicians [3,4], a mismatch between growing medical demands (for example, population ageing, which increases utilization of medical services [5]) and restricted supply (for example, intake rates to medical schools [4,6]) and retaining physicians in patient care until retirement age [4,7,8]. This study focuses on retention. Studies have found that the process of losing physicians from the workforce starts directly after graduation and persists throughout the physician career [9-11]. The problem is exacerbated by an increasing proportion of female physicians working part-time during childbearing age and the fact that female physicians are less likely than their male colleagues to have extended working hours [6,8,12]. To prevent actual leaving, reasons for leaving must be better understood. There is a long tradition of review studies focusing mainly on organizational turnover in employees [13], registered nurses [14] and physicians [15]. Less research has been conducted in recent decades on professional or occupational turnover intent, which is the focus of this review. To date, there is little evidence about the risk factors for physicians actually leaving patient care, although one study has shown that intention to leave (ITL) is a strong precursor of actually leaving [2]. Therefore, research on ITL could help identify a high-risk group for early intervention before leaving actually occurs.

As such, we aim to review the literature and summarize factors associated with the intention to leave direct patient care as reported by physicians. This is the first review study on this subject. Special attention is given to potential risk factors for the development of ITL and factors that can potentially be modified, such as working conditions and gender-specific workplace needs.

## Methods

### Review design

An integrative review was conducted according to the steps in the five-stage method recommended by Cooper [16]: formulation of the research problem, data collection, data evaluation, data analysis and presentation of the results.

### Search strategy and study selection

The first author conducted the database search. Relevant literature was extracted using a search of the following databases: MEDLINE, PsycINFO and Web of Science. Search terms were combinations of “intent(ion)(s) to” and “leave/withdraw/quit”, “leaving intention(s)”, combinations of “leaving” and “medicine/clinical care/patient care/the medical profession” and combinations of “quitting/withdrawing” and “intention(s)”. All search terms with more than 100 search results were restricted by AND (physician* OR doctor*). The search identified publications with at least one search term in the title or abstract. The earliest publication found was from 1992. As such, it was not necessary to limit the search period to select only recent publications on physician ITL direct patient care. The search covered all articles published prior to May 2014 in English or German. The references within the identified publications were also considered in order to find relevant literature not identified by the database search.

### Study selection criteria

Studies were included in the review if the original research was presented, the study population comprised postgraduate physicians, the study focus was the intention to leave direct patient care and the language was English or German. All studies that focused on turnover intentions such as intention to leave the current practice [17] or intentions to quit the current employment [18] were excluded from the review since there would be no net loss of physicians in the workforce in these cases.

### Search outcome

**Figure 1:**
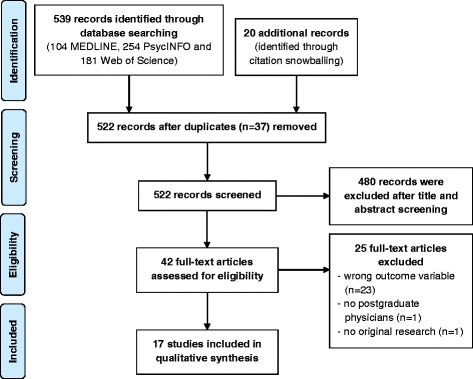
Flowchart on database search and study selection.

The first database search identified 539 hits (104 MEDLINE, 254 PsycINFO and 181 Web of Science), and snowballing procedures identified an additional 20 hits (Figure 1). After screening the titles and abstracts using the study selection criteria, 42 articles remained. Full-text examination of these articles reduced the number of studies included in this review to 17. Decisions to include or exclude studies were made by the first author under the supervision of the second author.

### Framework for analysis

Studies were synthesized using the following criteria: author information, sample, country, medical speciality, study design, career phase, research focus, study instruments, measurement of ITL, proportion of ITL and variables associated with ITL. To allow readers to evaluate the quality of the studies [19] included in the review, we provide additional details in Table 1 (research question, response rate, sample demographic, sample size) and Table 2 (research instrument). While we identified some weaknesses in the research instruments, which stemmed from the use of non-standard, self-designed questionnaires, we decided to analyse all studies for the sake of a broader literature review.

**Table 1:** **Summary of study characteristics and research focus**

**Author, year, country**	**Sample**	**Medical speciality**	**Design**	**Career phase**	**Research focus/question**
Bornschein et al. 2006 [21], Germany	Inpatient and outpatient care physicians in Munich 59% male Age: <35 = 1163 36–45 = 815 46–55 = 321 56+ = 136 Mean age: 38	Different specialities	*N* = 5,461/2,450 (RR 47%) Employed physicians Cross-sectional, Quantitative	All career stages	Assess the extent to which the German law on working hours is actually implemented in employed physicians
Davidson et al. 1998 [12], UK	Physicians qualified in the NHS in 1977 Mid 40s	Different specialities	*N* = 3,135/2,398 (RR 78.1%) Cross-sectional, Quantitative	Mid-career	Determine the career destinations by 1995 of physicians who qualified in 1977
Davidson et al. 2001 [29], UK	Physicians qualified in the NHS in 1974 Mean age: 48	Different specialities	*N* = 2,217/1,717 (RR 77.4%) Cross-sectional, Quantitative (open question)	Mid-career	Systematic information about retirement intentions and factors that influence them
Estryn-Behar et al. 2011 [23], France	French hospital physicians and *emergency physicians* 57.5% and *62.4% male* Age: <35 = 8.2% *and 25%* 35–44 = 33.8% *and 47%* 45–54 = 34.5% *and 23.5%* 55+ = 23.6% *and 4.5%*	Different specialities of French physicians and emergency physicians	*N* = 3,196 (RR 66%) from this a representative sample (*N* = 1,924) was randomly selected and compared to 538 emergency physicians Cross-sectional, Quantitative	All career phases	Analysis of working conditions, satisfaction and health on ITL and burnout for French physicians *with separate analysis for emergency physicians*
Estryn-Behar et al. 2011 [28], France	French hospital physicians 57.5% male Age: <35 = 8.2% 35–44 = 33.8% 45–54 = 34.5% 55+ = 23.6%	Different specialities	*N* = 3,196 (RR 66%) from this a representative sample (*N* = 1,924) was randomly selected Cross-sectional, Quantitative	All career phases	Analysis of the risk factors for burnout and intention to leave the profession *according to gender*
Fuss et al. 2008 [30], Germany	Physicians from two university hospitals in North Rhine-Westphalia 60.1% male Age: <34 = 126 35–44 = 98 45–54 = 44 55+ = 21 Mean age: 38.3	Different specialties	*N* = 761/296 (RR 38.9%) Cross-sectional, Quantitative	All career phases	Investigation of predictors for work interfering with family conflict
Goldacre et al. 1999 [37], UK	All physicians who qualified in the UK in 1996 and 1993 About 50% male Age: NS (junior physicians)	All specialties	In 1996, *N* = 3809/2926 (RR 76.8%) Longitudinal – comparison of 1996 and 1993 graduates Quantitative	Early career	To report the career intentions 1 year after qualification of physicians who qualified in the UK in 1996
Hann et al. 2010 [2], UK	Family physicians Male: NS Aged 50 and below	Family physicians	*N* = 2,000/1,174 (RR 67%) Longitudinal (5 years) – Secondary analysis, Quantitative	Mid-career	Does a family physician’s stated intention to leave his/her job predict whether or not he/she actually does leave?
Heponiemi et al. 2009 [27], Finland	Random sample of physicians 40.8% male Aged 25 to 65 Mean age: 45.9	All specialties	*N* = 5,000/2,841 (RR 57%) Final sample: 2650 Cross-sectional, Quantitative	All career phases	Examine whether job control moderates the association between stress indicators and intention to change profession
von dem Knesebeck et al. 2010 [31], Germany	Hospital physicians working in surgical fields 60.2% male Age: NS (920 residents and 391 consultants/senior physicians)	Surgery, gynaecology and obstetrics	*N* = 3,648/1,311 (RR 53%) Nationwide survey Cross-sectional, Quantitative	All career phases	Analyses of psychosocial stress in the workplace with the aid of the demand–control model, the effort–reward imbalance model and selected additional indicators
Moss et al. 2004 [20], UK	Graduates in 1999 at the end of the first postgraduate year <50% men Age of junior physicians: NS	Graduates from all medical schools	*N* = 4,221/2,727 (RR 64.6%) Analysed physicians = 1,326 Cross-sectional, Quantitative (open question)	Early career	Study reasons why junior physicians trained in UK consider leaving UK medicine
Ochsmann 2012 [24], Germany	Junior physicians in Bavaria in their first or second postgraduate year 42% men Mean age: 28.9	Different specialities	*N* = 1,494/792 (RR 53%) Analysed physicians = 637 Cross-sectional, Quantitative	Early career	Examine the association between workplace factors and thinking about leaving clinical care using a gender-stratified approach
Pachulicz et al. 2008 [26], US	Emergency physicians that completed residency in 1979,1984, 1988 and 1993 Over 80% men Age at 2004: primarily over the age of 45 with 6% over 65	Emergency physicians	1,269 participants in 2004 Longitudinal – three waves, Quantitative ITL was measured in 2004 (T3)	Mid-and late career	Objective and subjective career successes were hypothesized to mediate the relationships between socio-demographic variables, human capital indices, individual difference variables and organizational sponsorship as inputs and intention to leave medicine as the output variable
Rittenhouse et al. 2004 [33], US	Specialist physicians practising in large urban counties in California 89.8% male Age: <55 years = 62.8%, 55–64 years = 27.3% 65+ years = 9.9%	Different specialities	*N* = 1,492/978 (RR 66%) Longitudinal (3 years), Quantitative	All career phases	To validate physicians’ self-reported intention to leave clinical practice and the Masterfile practice status variable as measures of physician attrition and to determine predictors of ITL and actual departure from clinical practice
Scott et al. 2006 [25], UK	Representative sample of English and Scottish GPs 68% male Age in %: <35 = 0.14 35–39 = 0.19 40–44 = 0.21 45–49 = 0.18 50–54 = 0.17 55–59 = 0.09 60+ = 0.02	General practitioners	*N* = 4,421/2,781 (RR 62.9%) 1,968 questionnaires were available for analysis (44%). Cross-sectional, Quantitative	All career phases	Clarify the relationships between ITL, overall job satisfaction, domains of job satisfaction and personal and job characteristics by using a structural recursive model
Sibbald et al. 2003 [22], UK	Random sample general practitioner principals Male = 68.7% in 1998 and 70.6% in 2001 Age 1998 (2001): ≤35 = 113 (151) 36–40 = 187 (242) 41–45 = 195 (275) 46–50 = 125 (208) 51–55 = 102 (194) 56–60 = 53 (76) 61–65 = 15 (13) Mean age: 43.75 (44.35)	General practitioners	Sample 1998: 2,064/974 (RR 47%) Sample 2001: 2,000/1,332 (RR 67%) Final sample 790 in 1998 and 1,159 in 2001 Longitudinal, Quantitative	All career phases	Measure GPs’ intentions to quit direct patient care, to assess changes between 1998 and 2000 and to investigate associated factors, notably job satisfaction
Williams et al. 2001 [32], US	Clinically active physicians working in patient care in office or hospital settings aged <57 years 66.7% male Mean age: 43.7	Different specialities	*N* = 5,704/2,325 (adjusted RR 52%) Final sample 1,735 Cross-sectional, Quantitative	All career phases	Analysing a conceptual model of physician stress that explores its relationship with job satisfaction, physical and mental health and four types of withdraw intentions

*RR* response rate, *NS* not specified.

**Table 2:** **Research instruments, measure and percentage of physicians’ intention to leave direct patient care**

**Author, year, country**	**Sample size**	**Questionnaires/** **instruments/scales**	**Measurement of ITL**	**Percentage of ITL**
Bornschein et al. 2006 [21]^c^, Germany	2,450	Questionnaire about working hours and working conditions developed by authors	“Do you thinking about giving up medical practice in in- or out-patient care?” Three-point scale (“yes”, “I have thought about it”, “no”)	Overall ITL = 53.7%, 13.8% intended to quit their job and 39.8% had thought about quitting their job
Davidson et al. 1998 [12]^a^, UK	2,398	Questionnaire developed by authors	“Do you intend to practise in the NHS until normal retirement age?” Five-point scale (yes-definitely to no-definitely not)	1,714 physicians worked in the NHS: 22.9% of the NHS physicians did not intend to work until normal retirement age and 11.5% were undecided
Davidson et al. 2001 [29]^a^, UK	1,717	Questionnaire developed by authors	“Do you intend to practise in the NHS until normal retirement age for your current post?” Five-point scale (“yes, definitely” to “no, definitely not”)	1,427 physicians worked in the NHS: 25.2% of the NHS physicians definitely did not plan to continue until normal retirement age, 26% would probably not and 13.9% were undecided
Estryn-Behar et al. 2011 [23]^b^, France	538	Questionnaire from the NEXT study group adapted for physicians; focus on burnout, work–family conflict, satisfaction with pay scale, quality of teamwork, interpersonal relations within team, influence at work, quantitative demands, health	“How often during the course of the past year have you thought about giving up the medical profession?” Primarily over the age of 45-point scale (“never” to “every day”)	21.4% of the emergency physicians considered leaving the profession with answers of “sometimes in a month” or “more often”
Estryn-Behar et al. 2011 [28]^b^, France	1,924	Questionnaire from the NEXT study group adapted for physicians; focus on burnout, work–family conflict, satisfaction with pay scale, quality of teamwork, interpersonal relations within team, influence at work, quantitative demands, health	“How often during the course of the past year have you thought about giving up the medical profession?” Five-point scale (“never” to “every day”)	17.4% of French hospital physicians considered leaving the profession with answers of “sometimes in a month” or “more often”
Fuss et al. 2008 [30]^b^, Germany	296	Copenhagen Psychosocial Questionnaire (COPSOQ), work interfering with family conflict scale (WIF), items on hospital-specific work organization developed by the authors	“How often during the past 12 months have you thought about giving up your profession?” Five-point scale (“never” to “every day” – transformed to a range from 0 to 100)	Mean score = 18.6
Goldacre et al. 1999 [37]^a^, UK	2,926	Postal questionnaire developed by authors	“Apart from temporary visits abroad, do you intend to practise medicine in the UK for the foreseeable future?” If you did not answer “Yes, definitely”, are you: (a) considering practising medicine abroad? (b) considering leaving medicine but remaining in the UK? (c) considering leaving medicine and leaving the UK? Five-point scale (“yes, definitely” to “no, definitely not”)	9.4% of the UK-based qualifiers in 1996 and 6.8% of overseas-based respondents expressed the possibility that they might leave medicine (“yes, probably” and higher)
Hann et al. 2010 [2]^a^, UK	1,174	Data from the national survey of family physicians 2001 on ITL and job satisfaction and data from the annual census of physicians in the NHS for actually leaving	“Likelihood that they would leave direct patient care within the next 5 years” Five-point scale (“none” to “high”)	139 (11.8%) rated the likelihood of ITL within next 5 years as “likely” or “high”
Heponiemi et al. 2009 [27]^c^, Finland	2,650	Job control from Job Content Questionnaire, psychological distress from General Health Questionnaire, sleeping problems from Jenkins sleep problem scale	“If it were possible would you like to change to another profession with a similar salary?” Three response categories (“no”, “perhaps”, “yes”)	Not specified (in the regression analysis, the ITL measure was used as an ordinal variable which represents a higher level of ITL)
von dem Knesebeck et al. 2010 [31]^b^, Germany	1,311	Effort–reward–imbalance questionnaire, Job Content Questionnaire (JCQ), two items on the shift of stress from work to private life, two items for quality of patient care	“How often in the preceding 12 months they had thought of giving up their profession” Five-point scale (“never”, “several times a year, … month, … week”, to “every day”)	20.7% of the hospital physicians surveyed thought about giving up their profession at least a few times per month
Moss et al. 2004 [20]^c^, UK	1,326	Questionnaire developed by authors, main question “Specifying main reasons for considering leaving”	“Considering leaving medicine but remaining in the UK” and “considering leaving medicine and the UK” were grouped to “considering leaving medicine” Five-point scale (“yes, definitely” to “no, definitely not”)	42 of the 1326 physicians analysed (3.2%) were “probably not” or “definitely not” going to continue to practise medicine
Ochsmann 2012 [24]^b^, Germany	637	Questionnaire developed by authors on demographic background, medical education, current workplace factors and health-related questions	“During the last 12 months I thought about giving up clinical practice…” Five-point scale (“never” to “every day”)	52.3% thought about leaving clinical practice: 16 (2.5%) “every day”, 50 (7.8%) “a few times per week”, 98 (15.4%) “a few times per month”, 169 (26.5%) “a few times per year” and 304 (47.7%) “never”.
Pachulicz et al. 2008 [26]^a^, US	1,269	American Board of Emergency Medicine (ABEM) Longitudinal Study of Emergency Physicians (LSEP)	Intention to leave medicine: “Whether or not they hoped to leave medical practice for another career in the next 5 years” (1 = “yes”, 0 = “no”)	Not specified
Rittenhouse et al. 2004 [33]^a^, US	978	Survey of specialist physicians in urban California (1998); the American Medical Association (AMA) Physician Masterfile (2001); and direct ascertainment of physician practice status (2001) Main study items: career intention, career satisfaction, practice setting, practice experience	“Three years from now, do you think that you will be: (1) still practicing medicine and seeing patients; (2) still working in medicine but no longer seeing patients; (3) working in a career other than medicine; (4) retired.”	Intention to leave clinical practice = 8.8%: 5% intended to be still working in medicine but no longer seeing patients 3.8% intended to be working in a career other than medicine (12.8% intended to retire)
Scott et al. 2006 [25]^a^, UK	1,968	Questionnaire developed by authors	“Likelihood of leaving direct patient care within five years” (1 = “none”, 5 = “high”)	Mean score 2.15 (standard deviation 1.36)
Sibbald et al. 2003 [22]^a^, UK	790 in 1998, 1,159 in 2001	National postal surveys on overall job satisfaction, intention to leave direct patient care and physicians’ personal and practice characteristics	“Likelihood of leaving direct patient care (primary or hospital) within five years” Five-point scale (“none” to “high”)	Scores of 4 and 5 were determined as ITL Intending to quit direct patient care in the next 5 years rose from 14% in 1998 to 22% in 2001
Williams et al. 2001 [32]^a^, US	1735	Perceived stress scale, global job satisfaction, single item for physical health, mental health three-item measure (anxiety, depression and burnout)	Likelihood of “leaving the practice of direct patient care within 5 years” Five-point likelihood scale (3 = “moderate”)	18.5% of the physicians expressed a “moderate” or greater likelihood of leaving direct patient care

^a^Prospective measure.

^b^Retrospective measure.

^c^Universal measure.

## Results

### Study characteristics

The 12 cross-sectional and 5 longitudinal studies were published between 1998 and 2011 (Table 1). All studies used quantitative methods, and two included open questions on reasons behind ITL. The studies were conducted in different countries: the UK (seven studies), Germany (four), the US (three), France (two) and Finland (one). While most studies (11) investigated a wide range of specialities, 2 focused on emergency physicians, 3 on general practitioners (GPs) or family physicians, and 1 on surgical fields (surgery, gynaecology and obstetrics). All studies had mixed-gender samples, and the proportion of male physicians ranged from 40.8% to 89.9%. Most studies (10) covered all career phases, while 3 studied physicians in the early career phase, 3 studied mid-career physicians and 1 studied the mid- and late-career phases. The sample sizes in the studies reviewed ranged from 296 to 2926 (Table 2).

### Measuring ITL and the percentage of physicians intending to leave direct patient care

The reported percentage of physicians with ITL varied from 3.2% [20] to 53.7% [21] across the studies. Three measures were used to determine ITL: prospective measures asking physicians for the likelihood of leaving or intention to leave on a prospective time scale or until retirement (nine), retrospective measures asking for thoughts in the preceding 12 months (five) and universal measures without a time frame (three). The most commonly used prospective measure was “likelihood of leaving direct patient care within five years” (four); here, the percentage of physicians with ITL ranged from 11.8% to 22% (scores of 4 or 5 on a five-point Likert scale). The most frequently used retrospective measure was “how often during the past 12 months/course of the past year have you thought about giving up the profession/clinical practice” (five); here, the percentages ranged from 17.4% to 25.7% (scores of 3, 4 or 5 on a five-point Likert scale). Overall, the prospective percentages of physicians with ITL were slightly smaller than the retrospective percentages.

### Variables associated with ITL

Many different variables are associated with ITL. Therefore, similar explanatory factors were grouped under the following general terms: demographics, family or personal domain, working time and psychosocial working conditions, job-related well-being and career-related aspects.^a^

### Demographic variables

The studies reported significant associations of ITL with having children, ethnicity, medical speciality, age and gender. Physicians from ethnic minorities [22] and emergency physicians compared to hospital physicians [23] had a higher ITL. Female physicians working in paediatrics, anaesthesiology and surgery had lower ITL than those in internal medicine, while the same was true for male physicians in surgery and orthopaedics compared to those in internal medicine [24]. Lower ITL was also found in physicians with children under the age of 18 [22,25].

The demographic aspects of age and gender were discussed frequently in the studies. The next two paragraphs summarize these discussions.

### Age-specific findings

The impact of age on ITL was examined in eight studies. Three found null association with samples of physicians in their first or second postgraduate year [24] or primarily after the age of 45 [26] or primarily before the age of 45 [23]. Five studies with age ranges from postgraduate to retirement age found mixed results. Two studies found that younger physicians had a higher ITL [21,27]. The relative frequency of ITL at different ages was presented in one study [21], which indicated low ITL for very young physicians, the highest ITL for those aged 33–35 and decreasing ITL for physicians after the age of 50. Conversely, three studies indicated that older physicians had a higher ITL than those under the age of 35 [22,25,28].

### Gender-specific findings

Gender-specific analyses were conducted in seven studies. Two UK studies found that female physicians were more likely than their male colleagues to intend to remain in the National Health Service (NHS) [12,29]. Another study showed that male physicians with a partner and children under the age of 16 were less likely to indicate ITL [24].

Further gender-specific findings were identified for many of the explanatory factors. We describe these in the following after the general findings for each factor.

### Family or personal domain

Evidence of a connection between work–family conflict and ITL was observed in two studies. High work demands that interfered with family duties were associated with higher ITL [30]. Of all the reasons behind ITL, 26.1% belonged to the category “family reasons, time for leisure and other interests” and 19.6% belonged to the category “maintaining good health and wanting a healthy retirement” [29]. Sleeping problems were also associated with higher ITL [27].

Gender-specific findings showed that female physicians were more than twice as likely as their male colleagues to cite “family reasons, time for leisure and other interests” as a cause of ITL [29]. ITL was also higher for female hospital physicians with poor sleep quality [28], male emergency physicians and male hospital physicians who worried about making mistakes [23,28].

### Working time and psychosocial working conditions

Four studies reported an association between factors related to working time and ITL. Physicians with on-call duties and fewer breaks were more likely to indicate ITL [21]. One major reason for ITL was long working hours [20].

Three studies reported gender-specific findings. Female physicians reported higher ITL with 50 or more working hours each week [23], weekly overtime of 5 to 10 h [24] and long working hours [21]. Conversely, male physicians with fewer weekend duties reported lower ITL [24].

A variety of psychosocial working conditions were associated with ITL. A high degree of job control was associated with lower ITL; this factor also mitigated the positive effects of distress and sleeping problems on ITL [27]. Low quality of teamwork [28] and little or no work-related support from colleagues and superiors [24] increased ITL. Higher ITL was associated with high psychosocial stress (measured by effort–reward imbalance and job strain) [31], non-permanent positions [21] and dissatisfaction with the ability to choose method of working [25]. More than a third of physicians (36.5%) reported that the main reason for ITL was work-related pressure [29], while 35% cited poor working conditions in general [20].

Gender-specific results: ITL was higher for male emergency physicians with a low quality of teamwork [23], male hospital physicians reporting hostile relations with the administration [28] and men with fewer opportunities for postgraduate training [24]. Among women, ITL was higher for emergency physicians reporting monthly or more frequent harassment by superiors [23] and those with a low influence at work [23] and a lack of performance feedback [24].

### Job-related well-being

Several studies reported an association between job dissatisfaction and higher ITL [2,22,25,32]. The lack of job satisfaction was also regarded as an important reason behind ITL [20,29]. Another study found that job satisfaction mediated the link between perceived stress and ITL [32].

Four studies investigated mental health aspects. Higher ITL was linked with a high general burnout among emergency physicians [23]; patient-related burnout among hospital physicians [28]; poorer mental health as measured by depression, anxiety and burnout [32]; and high levels of psychosocial distress [27].

Gender-specific findings on high ITL were reported for female emergency physicians with high patient-related burnout [23] and female hospital physicians with high general burnout [28].

### Career-related aspects

Links were reported between career-related aspects such as job opportunities, salary and career satisfaction and ITL. Emergency physicians with job opportunities outside the health-care sector [23] and less salary progression reported higher ITL [26].

Gender-specific results for high ITL were found for male hospital physicians with job opportunities within and outside the health sector, women with a low salary satisfaction [28] and male emergency physicians with a lower career satisfaction [26].

### Associations between ITL and actually leaving

Two studies examined ITL and physicians actually leaving medical practice. A national survey of family physicians in England collected data (*N* = 1,174) on physicians’ ITL within the next 5 years [2]. The prevalence of ITL at the baseline survey was 11.8%, and the incidence of leaving direct patient care at the 5-year follow-up survey was 16.5%. The study showed that a higher ITL was associated with a greater likelihood of actually leaving.

In a survey of specialist physicians (*N* = 968) in California, data were collected on projected working status in 3 years time [32]. At the baseline, 8.8% of physicians reported ITL, and 10.6% (*N* = 101) of the initial sample was defined as having left clinical practice after 3 years.

## Discussion

In this review, we identified 17 studies of physicians’ intention to leave direct patient care from five countries and synthesized the study results. Variables associated with ITL were demographics, with age- and gender-specific findings, family or personal domain, working time and psychosocial working conditions, job-related well-being and career-related aspects. Three kinds of ITL measures were used: prospective, retrospective and universal. The share of physicians reporting ITL varied considerably between studies. The studies reviewed were diverse in terms of their aims, study design and instruments, making direct comparisons challenging.

### Critical consideration of the studies

Country-specific effects, such as different incentives and disincentives for leaving, could explain the diverse findings on age and ITL; for example, the studies with a high ITL among young physicians were from Germany [21] and Finland [27], while the studies reporting a high ITL among older physicians were from the UK [22,25] and France [28].

Different measures of ITL were used (Table 2) and different scale categories were determined for identifying ITL. Studies that determined ITL on a five-point Likert scale with scores of 3 or 4 [32] or on a three-point Likert scale with a score of 2 [21] should be interpreted with caution, as these scores included categories that indicated only a moderate ITL.

In the studies on ITL and actual leaving, the definition for actual leavers was not restricted to permanent leavers during the working-age period [2,33]. The results should be interpreted carefully in the context of these studies; for example, in the US study, most of the actual leavers were likely to have left due to normal retirement, as 9.9% of all physicians in the initial sample were aged 65 or over.

### Limitations of the review

Search terms including “turnover” were excluded from the review because of the broad definition of turnover intentions [34] and the problem of separating turnover intention from intention to leave a job and intention to leave the profession. As our aim was to identify risk factors for intention to leave direct patient care, we decided not to include turnover intention in order to obtain rigorous results on the intention to leave direct patient care.

Decisions to include or exclude studies were made by two authors, whereas the database search was conducted by just one author. Therefore, the validity of the initial records included was not verified by a second author.

In two studies [12,29], Davidson investigated the intention to practise in the NHS until normal retirement age. In the UK, it is possible to work as a physician in the NHS or in private practice. This makes it possible for physicians to leave the NHS and switch to private practice. In the study by Davidson [29], the analysis of the reasons behind intending to leave before retirement age makes it clear that changing to private practice was not a reason for considering early retirement. Davidson [12] researched a different cohort of physicians from the same database, but the reasons behind considering early retirement are not known for these physicians. This last study by Davidson [12] was not excluded from the review, as the two cohorts differed by just 3 years. Furthermore, most physicians in the UK tend to work predominantly for the NHS and take only part-time work in private practice, if at all. This highlights that retiring from the NHS early and shifting to private practice may not be an attractive option, as this leaves too little time to amortize the additional investment and operating costs.

This shows that the differences in health-care systems, working environments and physician career systems in the countries investigated could lead to research bias. Further research is needed for a systematic examination of the prevailing conditions in each of these countries to reduce this potential bias. Another limitation concerns the small number of countries (Germany, the UK, France, Finland and the US) included in this review and the absence of low- and middle-income countries. This is partly due to the requirement that articles be in English or German, which introduced language bias.

### Recommendations for future research

Unfortunately, we were unable to identify a relevant study that used qualitative methods to conduct an in-depth analysis of the personal perspectives of and context behind physicians with ITL [35]. In-depth qualitative analysis could provide an overall picture of reasons behind ITL and lead to the development of a standard and rigorously psychometrically tested instrument for this research topic. Most of the studies reviewed used self-developed instruments.

Secondly, attention should be paid to how ITL is measured. It is not clear which kind of ITL measure has better explanatory power when it comes to linking risk factors and actual leaving. This methodology decision to use a prospective, retrospective or universal ITL measure is an issue for further research.

Thirdly, studies in the review had diverse aims and study designs, resulting in different sets of variables being considered in this analysis. Therefore, no single study included all of the job and personal variables that have been attributed to ITL. To present the results of similar factors together, we grouped the key findings into several categories (see the “Results” section above). In reality, all of these factors are correlated and are difficult to differentiate from each other. According to earlier findings [25], overall job satisfaction combined with job and personal characteristics was better able to predict the outcome than any single variable set. In future, a wide range of all relevant predictors should be considered in ITL analyses.

Finally, gender-specific analyses were conducted in seven studies and revealed that men and women often had different risk factors under similar circumstances; for example, in terms of psychosocial working conditions, higher ITL was most closely associated with a low quality of teamwork for men and a low influence at work for women. This makes it clear that men and women have different workplace needs. Deeper exploration of gender differences is warranted on this topic.

## Conclusions

This integrative review provides an overview of postgraduate physicians’ intentions to leave direct patient care. Early intervention on risk factors for ITL could prevent physicians from actually leaving. Based on the findings of this current review, starting points for intervention could focus on work–family conflict [29,30], especially for women [29]. Factors related to working time also appear to be important and, again, particularly for women [21,23,24]. Health-care managers and policy-makers should consider working time and work–family conflict as priorities when it comes to retaining physicians. These issues become even more critical as female physicians form an increasingly large share of the physician workforce [4-6]. There is also much evidence to indicate that job stress caused by adverse psychosocial working conditions increases ITL. Health-care managers, hospital administrators and policy-makers could address this ITL driver by improving psychosocial working conditions for the medical profession. The success of such interventions has been observed with long-term effectiveness for a wide range of psychosocial work factors and well-being among health-care professionals [36].

## Endnotes

^a^Rittenhouse et al. [33] include retirement intentions in the ITL outcome in their causal analyses (Table 2, ITL = categories 2, 3 and 4). Therefore, these results are not included in this section of our review.
